# First Report of *Aeromonas veronii* as an Emerging Bacterial Pathogen of Farmed Nile Tilapia (*Oreochromis niloticus*) in Brazil

**DOI:** 10.3390/pathogens12081020

**Published:** 2023-08-08

**Authors:** Sandie Bispo dos Santos, Miguel Fernandez Alarcon, Anelise Stella Ballaben, Ricardo Harakava, Renata Galetti, Mateus Cardoso Guimarães, Mariene Miyoko Natori, Leonardo Susumu Takahashi, Ricardo Ildefonso, Marco Rozas-Serri

**Affiliations:** 1 Pathovet Labs, Ribeirão Preto 14025-020, Brazil; sandie.barbosa@pathovet.cl (S.B.d.S.); miguel.fernandez@pathovet.cl (M.F.A.); renata.galetti@pathovet.cl (R.G.); mateus.cardoso@pathovet.cl (M.C.G.); mariene.natori@pathovet.cl (M.M.N.); ricardo.ildefonso@pathovet.cl (R.I.); 2 Faculdade de Ciências Farmacêuticas de Ribeirão Preto, Universidade de São Paulo, Ribeirão Preto, São Paulo 14040-020, Brazil; aneliseballaben@usp.br; 3 Instituto Biológico/IB, São Paulo 04016-035, Brazil; ricardo.harakava@sp.gov.br; 4 Departamento de Produção Animal, Faculdade de Ciências Agrárias e Tecnológicas, Universidade Estadual Paulista, Dracena, São Paulo 17900-000, Brazil; leonardo.takahashi@unesp.br; 5 Pathovet Labs, Puerto Montt 5550000, Chile

**Keywords:** Nile tilapia, Motile Aeromonas Septicemia, MAS, *Aeromonas veronii*, antimicrobial-resistant

## Abstract

Brazil is one of the world’s leading producers of Nile tilapia, *Oreochromis niloticus*. However, the industry faces a major challenge in terms of infectious diseases, as at least five new pathogens have been formally described in the last five years. *Aeromonas* species are Gram-negative anaerobic bacteria that are often described as fish pathogens causing Motile Aeromonas Septicemia (MAS). In late December 2022, an epidemic outbreak was reported in farmed Nile tilapia in the state of São Paulo, Brazil, characterized by clinical signs and gross pathology suggestive of MAS. The objective of this study was to isolate, identify, and characterize in vitro and in vivo the causative agent of this epidemic outbreak. The bacterial isolates were identified as *Aeromonas veronii* based on the homology of 16S rRNA (99.9%), *gyrB* (98.9%), and the *rpoB* gene (99.1%). *A. veronii* showed susceptibility only to florfenicol, while it was resistant to the other three antimicrobials tested, oxytetracycline, enrofloxacin, and amoxicillin. The lowest florfenicol concentration capable of inhibiting bacterial growth was ≤0.5 µg/mL. The phenotypic resistance of the *A. veronii* isolate observed for quinolones and tetracycline was genetically confirmed by the presence of the *qnrS2* (*colE* plasmid) and *tetA* antibiotic-resistant genes, respectively. *A. veronii* isolate was highly pathogenic in juvenile Nile tilapia tested in vivo, showing a mortality rate ranging from 3 to 100% in the lowest (1.2 × 10^4^) and highest (1.2 × 10^8^) bacterial dose groups, respectively. To our knowledge, this study would constitute the first report of highly pathogenic and multidrug-resistant *A. veronii* associated with outbreaks and high mortality rates in tilapia farmed in commercial net cages in Brazil.

## 1. Introduction

Brazil is the fourth-largest producer of Nile tilapia, *Oreochromis niloticus,* and the country with the highest growth potential in the world [1]. However, being an emerging animal production industry, Brazilian tilapia culture faces several challenges, at least from the biological point of view, the increased susceptibility of fish to infectious diseases, including the lack of normative regulation for health management with the mission to optimize disease prevention and control.

Consequently, in addition to the recent first report of infectious spleen and kidney necrosis virus (ISKNV) in tilapia farmed in Brazil [2], at least four emerging bacterial pathogens have been described just in the last four years: *Plesiomonas shigelloides* [3], *Aeromonas jandaei* [4], *Klebsiella pneumoniae* [5], and *Lactococcus petauri* [6]. Although several species of the genus *Aeromonas*, e.g., *A. hydrophila* and *A. jandaei*, have been described in farmed tilapia in Brazil, *A. veronii* has not been reported to date.

*Aeromonas* species are Gram-negative anaerobic bacteria that are often described as fish pathogens causing Motile Aeromonas Septicemia (MAS) [7]. MAS is a severe systemic disease that affects fish in both freshwater and saltwater environments and can cause high mortality rates [8]. The gross pathology of MAS is characterized by reddened flippers, diffuse hemorrhages in the skin and anus, exophthalmia, and abdominal swelling. Internal findings include bloody ascites, diffuse hemorrhages in the intestine and skeletal muscle, and swollen and friable kidneys and spleen [7,8]. Several studies have isolated and characterized specifically *A. veronii* from tilapia [7,9,10,11], rainbow trout, *Oncorhynchus mykiss* [12], channel catfish, *Ictalurus punctatus* [13,14], Chinese longsnout catfish, *Tachysurus dumerili* [15], crucian carp, *Carassius auratus gibelio* [16], common carp, *Cyprinus carpio* [17], Pond loach, *Misgurnus anguillicaudatus* [18], and dark sleeper *Odontobutis potamophila* [19]. Importantly, *Aeromonas* species cause a wide range of diseases in humans, such as gastrointestinal tract syndromes, wound and soft tissue infections, and blood-borne dyscrasias, among others [20], and could act as important antibiotic-resistant gene (ARG) vectors for clinically relevant fish pathogens that share the same breeding sites [21].

In late December 2022, an epidemic outbreak was reported in farmed Nile tilapia in the state of São Paulo, Brazil, characterized by clinical signs and gross pathology suggestive of MAS. This article presents the results of the study that aimed to: (a) isolate and identify the putative causative bacterial agent of the mortality outbreak by conventional isolation methods, DNA sequencing of specific 16S rDNA targets, and housekeeping genes encoding the DNA gyrase B subunit (*gyrB*) and RNA polymerase B subunit (*rpoB*), and phylogenetic analysis; (b) assess antimicrobial susceptibility, minimum inhibitory concentration (MIC), and ARGs on the causative agent to recommend optimal disease treatment under field conditions; and (c) confirm Koch’s postulates and evaluate the pathogenicity of the causal agent by an in vivo experimental infection challenge. In the present study, we have described the isolation, characterization, and virulence of *A. veronii* causing MAS in Nile tilapia in Brazil.

## 2. Material and Methods

### 2.1. Fish Sampling

In late December 2022, an outbreak of mortality was reported in net-cage-farmed Nile tilapia in the state of São Paulo, Brazil, characterized by gross pathology consistent with bacterial hemorrhagic septicemia and a cumulative mortality rate reaching 50% in one month. Diseased fish showed erratic swimming, exophthalmos, ocular opacity, skin ulcers, and ascites. Ten moribund fish weighing 25–35 g were sampled from six net cages and transported alive in bags with water in a polyethylene box and under refrigeration temperature between 2 and 6 °C to Pathovet Labs, Ribeirão Preto, São Paulo, Brazil. All fish, tested in the tissue pool (spleen, kidney, and liver) by PCR, were negative for the main enzootic pathogenic bacteria in this geographical area of tilapia farming in Brazil, but positive for ISKNV (average CT 23.14). However, no clinical signs or gross pathology attributable to ISKNV were observed in the fish tested in the field, and no histopathological lesions attributable to ISKNV were observed.

### 2.2. Bacterial Isolation and Identification

Each fish was subjected to morphometric examination and inspected externally and internally by anatomopathological examination. Samples of different tissues were collected and preserved for further bacteriological and virological analysis. Spleen and kidney samples were aseptically seeded on Brain Heart Infusion (BHI) agar supplemented with 5% defibrinated sheep blood (Heel do Brasil Biomedica Ltd.a., São José dos Pinhais, PR, Brazil) for the isolation of bacteria of the genera *Streptococcus*, *Lactococcus*, *Aeromonas*, *Edwardsiella*, *Plesiomonas,* and *Pseudomonas*. The plates were immediately incubated at 28 °C in a BOD incubator (Tecnal Company, São Paulo, Brazil) for 48 h. Based on dominance, definite colony morphology, and Gram stain observation results, suspect isolates were picked, purified by repeated streaking on BHI, and maintained on BHI at 8 °C, and as glycerol stock at −80 °C for further characterization. Bacterial colonies cultured were negative for PCR *A. hydrophila*, *S. agalactiae*, *E. tarda*, and *E. anguillarum*.

### 2.3. Molecular Characterization and Phylogenetic Analysis

Genomic DNA was extracted using a MagMAX™ CORE Nucleic Acid Purification kit (Thermo Fisher Scientific Inc., São Paulo, SP, Brazil) according to the manufacturer’s recommendations. Then, tissue was homogenized in PBS solution (pH 7.4), agitated in L-Beader 24 disruptor (Loccus do Brasil Ltd.a., Cotia, SP, Brazil), centrifuged at 1000× *g* for 2 min, and automatically extracted using KingFisher™ Duo prime system equipment (Thermo Fisher Scientific Inc., São Paulo, SP, Brazil) according to the manufacturer’s protocol. PCR reactions consisted of 10 µL of 5X PCR buffer, 1 µL of forward primer (10 µM), 1 µL of reverse primer (10 µM), 1 µL of dNTPs (10 mM), 0.2 µL of GoTaq DNA polymerase (5 U/µL Promega), and 36.8 µL of autoclaved MilliQ H_2_O.

PCR was performed for amplification of the bacterial 16S rRNA using universal primers fD1 (5’-AGAGTTTGATCCTGGCTCAG-3’) and rP1 (5’-ACGGTTACCTTGTTACGACTT-3’) as previously described by Weisburg et al. [22]. In addition, partial *rpoB* gene was amplified using in-house designed primers rpoB-F (5′-CGTCTGTCTCTGGGYGATCT-3′) and rpoB-R (5′-CCGCCTGACGTTGCATGT-3′) and partial *gyrB* gene was amplified using primers gyrB-F (5′-GTVCGTTTCTGGCCVAG-3′) and gyrB-R (5′-GCNGGRTCYTTYTCYTGRCA-3′).

Amplification was performed in a T100 Thermal Cycler (Bio-Rad Laboratories, Hercules, CA, USA) using the following program for 16S rRNA: initial denaturation at 94 °C for 4 min, 40 cycles of 94 °C for 30 s, 60 °C for 30 s, 72 °C for 90 s, and final extension at 72 °C for 4 min. For *rpoB* and *gyrB*, the annealing temperature was 54 °C and the extension time was 45 s. PCR products were electrophoresed through 0.8% agarose/tris-borate EDTA buffer and visualized by staining with ethidium bromide (100 ng/mL), purified using polyethylene glycol precipitation [23], and submitted to sequencing reaction by the chain-termination method [24]. Briefly, the reaction consisted of 5 µL of PCR product, 1 µL of Big Dye 3.1 reagent (Applied Biosystems, Waltham, MA, USA), 1.5 µL of dilution buffer, 0.3 µL of either forward or reverse primers (10 µM), and 2.2 µL of autoclaved MilliQ H2O. The reaction was performed in a T100 Thermal Cycler (Bio-Rad Laboratories, Hercules, CA, USA) with the following program: initial denaturation at 95 °C for 1 min followed by 25 cycles of 95 °C for 5 s and 60 °C for 4 min.

DNA sequencing reaction products were precipitated by the addition of 40 µL of 75% isopropanol, followed by centrifugation at 12,000× *g* for 10 min. Another 100 µL of 75% isopropanol was added and again centrifuged at 12,000× *g* for 5 min. After discarding the supernatant, the pellet was dried and resuspended in 10 µL formamide and denatured at 95 °C for 2 min. Sequencing was performed on a 3500XL Genetic Analyzer capillary sequencer (Applied Biosystems, Waltham, MA, USA).

DNA sequences from bacterial isolates were compared to sequences from type specimens deposited in the Genbank using Blastn. Thereafter, 16S rRNA (1426 nt), *rpoB* (493 nt), and *gyrB* (652 nt) sequences were concatenated, aligned by ClustalW, and submitted to phylogenetic analysis using MEGA 11 [25]. The best-fit substitution model found was Tamura-Nei (TN93) using discrete Gamma distribution (+G) and assuming that some sites are evolutionarily invariable (+I). The Maximum-Likelihood phylogenetic tree was constructed using a bootstrap of 1000 repetitions.

### 2.4. Antibiotic Susceptibility and Minimum Inhibitory Concentration (MIC)

The susceptibility of the bacterial isolate to antibiotics was assessed by the standard disk diffusion method [26]. The four most used antibacterial agents to treat bacterial diseases in tilapia raised in Brazil (enrofloxacin, oxytetracycline, amoxicillin, and florfenicol) were tested according to the manufacturer’s protocol and recommendations. Complementarily, the lowest concentration of each antibiotic capable of totally inhibiting bacterial growth was estimated using the guidelines of the European Committee for Antimicrobial Susceptibility Testing (EUCAST) [27] and the Brazilian Committee for Antimicrobial Susceptibility Testing (BrCast) (https://brcast.org.br/wp-content/uploads/2022/07/Tabela-pontos-de-corte-clinicos-BrCAST-v1-mar-2021.pdf, accessed on 3 May 2023).

### 2.5. Molecular Detection of Resistance Genes

ARGs encoding extended-spectrum β-lactamases—ESBLs (*bla*_CTX-M_-groups 1, 2, 8, 9, and 25) [28], carbapenemases (*bla*_NDM_, *bla*_IMP_, *bla*_VIM_, *bla*_KPC_, and *bla*_OXA-48_) [29], plasmid-mediated quinolone resistance determinants (*qnrA*, *qnrB, qnrC, qnrD, qnrS,* and *colE*) [30,31,32,33], and tetracycline resistance (*tetA, tetB, tetC, tetD, and tetE*) [34] were screened by conventional PCR (Table 1). PCR was performed with a final volume of 25µL being added: 60 ng of genomic DNA, 0.2 mM of each of the four nucleotides, 2.5µL of concentrated 10X PCR buffer, 2.0 mM of MgCl2 solution, 0.625U of Taq DNA polymerase (Thermo Fisher Scientific, Waltham, MA, USA), 21 pmol of each of the primers (Invitrogen Life Technologies, Carlsbad, CA, USA), and ultrapure water (q.s.p. 25 µL, Sigma-Aldrich, Barueri, SP, Brazil). The PCRs were carried out in the Eppendorf 5331 Gradient MasterCycler PCR System (Eppendorf, Hamburg, Germany) using the following program: 94 °C for 5 min for initial denaturation followed by 30 cycles at 94 °C for 1 min, and 72 °C for 1 min. Finally, a single final extension step at 72 °C for 10 min.

### 2.6. Experimental Challenge

#### 2.6.1. Preparation of Bacterial Inoculum

To prepare the inocula, the *A. veronii* isolate was seeded in BHI agar culture medium (Heel do Brasil Biomedica Ltd.a., São José dos Pinhais, PR, Brazil) and incubated in a BOD-type incubator for 24 h at 28 °C. Pure colonies were transferred to tubes containing 30 mL of BHI broth medium and incubated at 28 °C for 22 h. Then, the tubes were centrifuged at 4 °C at 3200× *g* for 10 min. The pellet was washed three times with saline solution and resuspended in 6 mL of sterile saline solution. The optical density of the bacterial solution was measured in a densitometer obtaining a stock solution at 3 × 10^9^ CFU/mL. Subsequently, serial 10-fold dilutions were performed in 0.85% saline solution to the concentrations of 1.2 × 10^1^-to-1.2 × 10^8^ CFU/mL and seeded onto BHI medium plates and incubated in a BOD-type incubator for 24 h at 28 °C.

#### 2.6.2. Challenge Trial

The study was carried out at the facilities of Pathovet Labs, Ribeirão Preto, SP, Brazil. Three hundred (300) apparently healthy Nile tilapia (weighing 3 g) were obtained from a tilapia commercial farm in São Paulo and kept in a 500 L capacity tank containing 300 L treated water at a temperature of 27.2 °C ± 1.05. A sample of thirty (30) fish were tested for the presence of bacterial and viral pathogens endemic to farmed tilapia in Brazil using PCR assays and bacteriological cultures prior to entry into the facility. Subsequently, a total of two hundred and forty (240) fish were randomly distributed in groups of ten (10) individuals in twenty-four glass aquaria (70 L) for acclimatization for twenty days. After this time, fish were randomly selected, anesthetized with a solution of 1 g per 10 L of clove oil [35], subjected to morphometric examination, and injected intraperitoneally (I.P.) with 0.1 mL of bacterial suspension with five different concentrations of *A. veronii* (1.2 × 10^4^, 1.2 × 10^5^, 1.2 × 10^6^, 1.2 × 10^7^, and 1.2 × 10^8^ CFU/mL). Thereby, forty fish were inoculated with each dilution and randomly distributed into four aquaria of ten fish (quadruplicate). Forty other fish were injected with sterile saline and distributed in the same way as a control group. Morbidity and mortality were observed in all fish groups for fifteen days and the moribund and/or freshly affected dead specimens were used for the re-isolation of *A. veronii*. Continued aeration was provided with an exchange of 50% of the water daily. The water quality parameters during the experimental challenge trials were as follows: temperature at 27.2 °C ± 1.05, pH at 6.82 ± 0.35, dissolved oxygen at 4.79 ± 1.11 mg/L, ammonia at 0.44 ± 0.10 mg/L, and nitrite at 0.45 ± 0.27 µg/L. Clinical signs, gross pathology, and mortality were recorded three times daily for fifteen days post-inoculation (dpi). Moribund fish and/or fresh dead fish from each aquarium were analyzed by the microbiological and molecular methods described earlier in this section.

#### 2.6.3. Histopathology

Liver, kidney, and spleen samples were collected from twenty-six surviving fish at the end of the trial (six fish from the control, 10^4^, 10^5^, and 10^6^ dilution groups, and two fish from the 10^7^-dilution group) and fixed in 10% phosphate-buffered formalin for at least 24 h. Subsequently, the samples were dehydrated using a graded alcohol series and processed for histological examination. Sections (5 μm) of the tissues were stained with hematoxylin and eosin (H&E) and observations were made with a light microscope (Leica DM1000, Hamburg, Germany). 

## 3. Results

### 3.1. The Outbreak in Farmed Nile Tilapia in Brazil Was Caused by A. veronii

**Figure 1:**
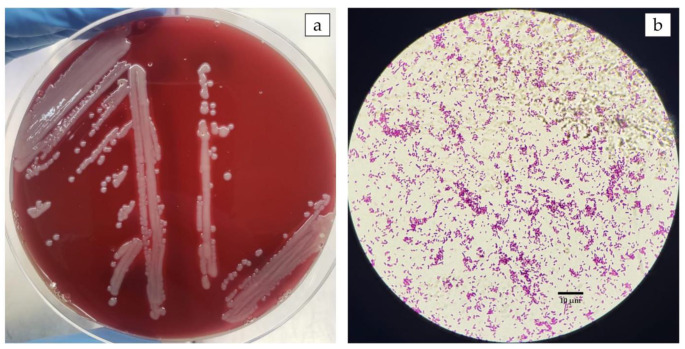
Morphological characteristics of *Aeromonas veronii* isolated from a disease outbreak in Nile tilapia farmed in Brazil. (**a**) Bacterial culture on BHI agar from diseased fish tissues collected in the field. Presence of pinhead-sized, semitransparent, yellowish, flattened colonies. (**b**) Gram-negative bacilli under a light microscope (Scale bar 10 μm).

**Figure 2:**
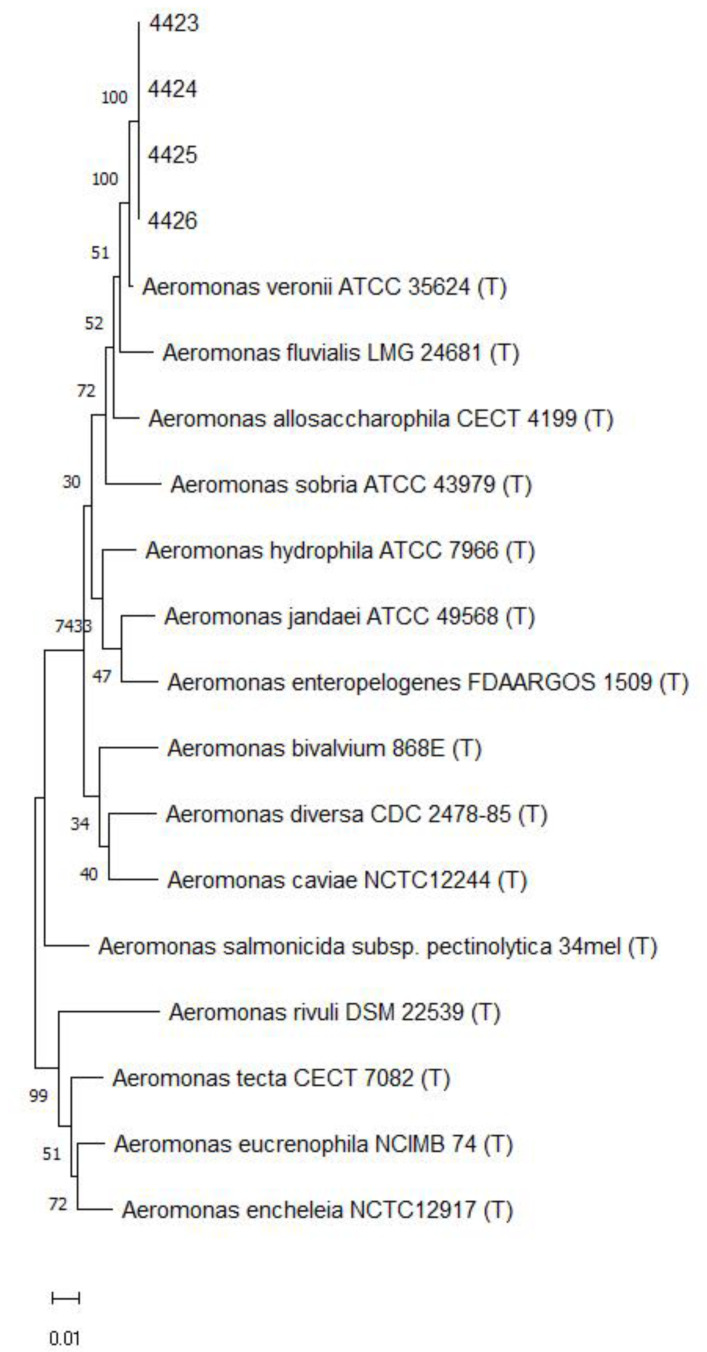
A phylogenetic tree constructed based on concatenated *16S rRNA, gyrB,* and *rpoB* gene sequences of four clinical isolates from this study (4423, 4424, 4425, 4426) and from type strains (T) of *Aeromonas* spp. retrieved from the Genbank. The Maximum likelihood method was used with the Tamura-Nei model with Gamma distribution and allowing some sites to be evolutionarily invariable. The support level in percentage, after 1000 repetitions, is indicated next to each branch.

Following the bacterial culture of diseased fish tissues collected in the field, the presence of pinhead-sized, semitransparent, yellowish, flattened colonies was detected. Gram-negative bacilli were confirmed (Figure 1). The species and genus of the bacterial isolate were confirmed by the sequencing of the 16S rRNA (~1.5 kb), *rpoB* (493 bp), and *gyrB* (652 bp) gene amplicons obtained from DNA extracted from a pure colony of isolates. The sequences obtained showed 99.9% similarity with the partial sequence of the 16S rRNA gene of the *Aeromonas veronii* strain ATCC 35,624 (GenBank access number NR 118947). Likewise, the sequences obtained from the *rpoB* and *gyrB* genes corroborate the identification of the isolates as *A. veronii* with 99.1% and 98.9% similarities, respectively, with sequences of the *A. veronii* type strain. Sequences were submitted to GenBank (National Center for Biotechnology Information, US National Library of Medicine, Bethesda, MD) under the accession numbers OR227081-227084 (16S rRNA), OR236713-236716 (*rpoB*), and OR236717-236720 (*gyrB*). A phylogenetic tree based on the alignment of concatenated nucleotide sequences of 16S rRNA, *rpoB*, and *gyrB* genes (Figure 2) clustered the isolates of the current study with the *A. veronii* type strain ATCC 35624.

### 3.2. The Isolate of A. veronii Showed Multidrug Resistance to the Most Used Antibacterial Drugs in Nile Tilapia Farmed in Brazil

**Figure 3:**
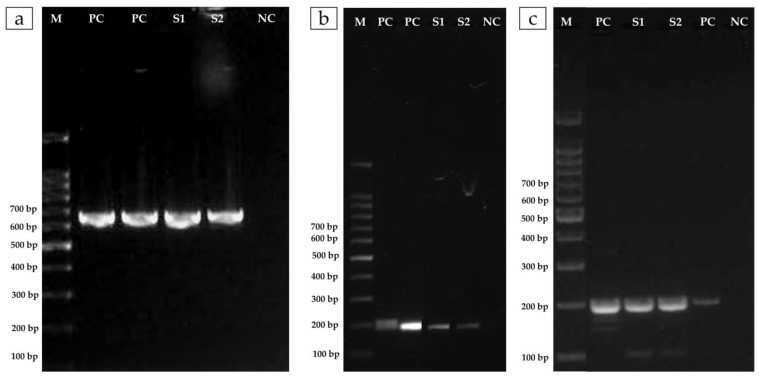
Gel electrophoresis showing amplification products for antibiotic resistance genes in *A. veronii* strains isolated from farmed Nile tilapia naturally challenged in field conditions, and re-isolated from Nile tilapia experimentally challenged: (**a**) Quinolone resistance *qnrS* gene (~680 bp) Line 1: DNA ladder, 100 bp, Lines 2 and 3: positive controls, Lines 4 and 5: *A. veronii* isolates, Line 6: negative control; (**b**) *colE* plasmid (~190 bp) Line 1: DNA ladder, 100 bp, Lines 2 and 3: positive controls, Lines 4 and 5: *A. veronii* isolates, Line 6: negative control; and (**c**) Tetracyclines *tatA* gene (~210 bp), Line 6: DNA ladder, 100 bp, Lines 1 and 4: positive controls, Lines 2 and 3: *A. veronii* isolates, Line 5: negative control.

*A. veronii* showed sensitivity only to florfenicol, while it was resistant to the other three antimicrobials tested, oxytetracycline, enrofloxacin, and amoxicillin (Table 2). The MIC observed was 32 µg/mL for oxytetracycline, 4 µg/mL for enroflorxacin, and ≥256 µg/mL for amoxicillin. As shown in the antibiogram, the lowest concentration capable of inhibiting bacterial growth was observed for florfenicol (≤0.5 µg/mL) (Table 2). Complementarily, the phenotypic resistance of the *A. veronii* isolate observed for quinolones and oxytetracycline was genetically confirmed by the presence of the *qnrS-colE* and *tetA* genes, respectively (Figure 3).

### 3.3. A. veronii Showed High In Vivo Pathogenicity and Koch’s Postulates Were Fulfilled

**Figure 4:**
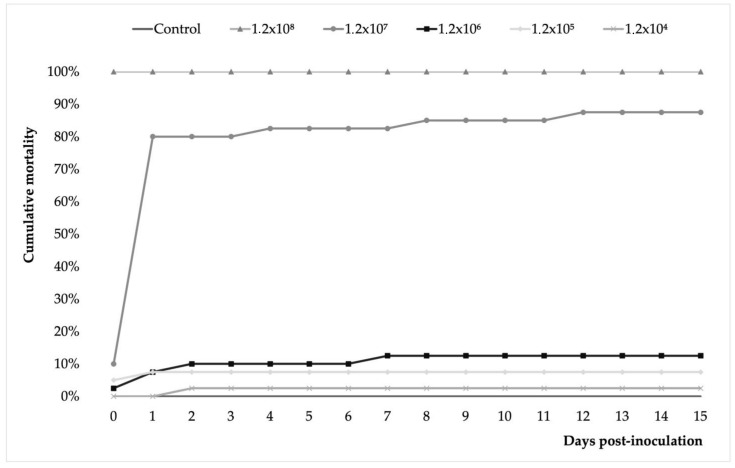
Cumulative mortality during the 15-day I.P. challenge trial conducted on juvenile Nile tilapia inoculated with 0.1 mL of *A. veronii* bacterial solution at different concentrations (1.2 × 10^3^–1.2 × 10^8^) and a control group injected with 0.1 mL of saline. Each point on the curves shows the mean of four replicates per treatment and control group. The results showed that the *A. veronii* isolate tested was pathogenic in juvenile Nile tilapia with dose-dependent mortality rates and a degree of pathogenicity.

The results showed that the *A. veronii* isolate tested was pathogenic in juvenile Nile tilapia with dose-dependent mortality rates and a degree of pathogenicity. The mortality rate ranged from 3 to 100% in the lowest (1.2 × 10^4^) and highest (1.2 × 10^8^) in the bacterial dose groups (Figure 4). In the 1.2 × 10^7^ group, cumulative mortality reached 10% before the first 24 h after inoculation. Thereafter, cumulative mortality rapidly reached 80% at 48 dpi and continued with a milder increase to 83% at 4 dpi, 85% at 8 dpi, and 88% at 12 dpi and until the end of the trial. Fish in the 1.2 × 10^6^ group showed a similar pattern of cumulative mortality but with significantly lower values. Like this, cumulative mortality registered 10% at 48 h post-inoculation and 13% at 7 dpi, a rate that remained unchanged until the end of the challenge. No mortality was recorded in the control group.

**Figure 5:**
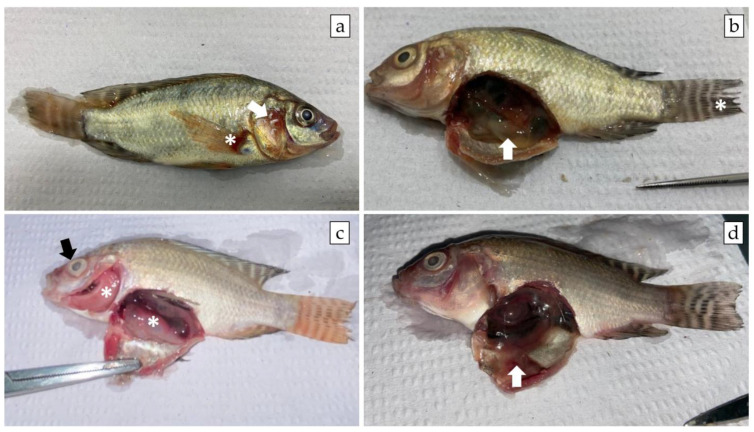
Gross pathology findings in juvenile Nile tilapia experimentally infected by *A. veronii* isolate obtained and characterized from a disease outbreak in a commercial net cage farm in the state of Sao Paulo, Brazil. (**a**) Opercular hemorrhage (white arrow) and hemorrhage at the base of the fins (white asterisk) (Group 1.2 × 10^6^); (**b**) Intestine filled with transparent fluid (white arrow) and ragged edges to the caudal fin (white asterisk) (Group 1.2 × 10^5^); (**c**) Ocular opacity (black arrow), pale gills, and visceral organs (white asterisk) (Group 1.2 × 10^5^); (**d**) Ascites (Group 1.2 × 10^4^).

**Figure 6:**
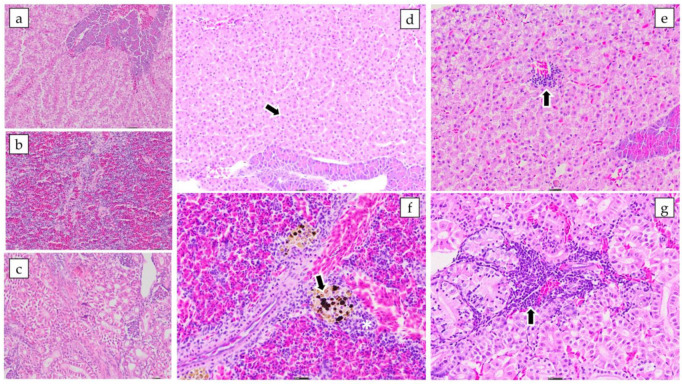
Microphotographs of juvenile Nile tilapia tissues experimentally infected with a pathogenic strain of *Aeromonas veronii*. Haematoxylin-eosin staining. Microscopic tissue changes were often mild to moderate and particularly in infected fish with lower bacterial loads: (**a**) No microscopic changes in the liver. Scale bar 50 μm; (**b**) No microscopic changes in the spleen. Scale bar 20 μm; (**c**) No microscopic changes in the kidney. Scale bar 20 μm; (**d**) Liver (Group 1.2 × 10^6^). Diffuse vacuolar depletion (arrow). Scale bar 20 μm; (**e**) Liver (Group 1.2 × 10^5^). Focal mononuclear inflammatory infiltrate (arrow). Scale bar 20 μm; (**f**) Spleen (Group 1.2 × 10^5^). Hyperplasia of melano-macrophage (arrow) centers and lymphoid hyperplasia (white asterisk). Scale bar 20 μm; and (**g**) Kidneys (Group 1.2 × 10^4^). Focal interstitial leukocyte infiltrate (arrow). Scale bar 20 μm.

The diseased fish showed clinical signs and gross pathology characteristic of MAS such as hemorrhage at the base of the fins, opercular hemorrhage, and ascites (Figure 5). Microscopically, tissue changes were often mild to moderate, especially in surviving fish infected with lower bacterial doses, as fish inoculated with higher doses died per- acutely. Briefly, the main histopathological changes recorded were hyperplasia of melano-macrophage centers and lymphoid hyperplasia in the spleen, diffuse vacuolar depletion and focal mononuclear inflammatory infiltrate in the liver, and focal interstitial leukocyte infiltrate in the kidney (Figure 6). Pure bacterial colonies resembling *Aeromonas* spp. were recovered from the liver, kidney, and spleen of dead and moribund fish from all groups and were finally identified as *Aeromonas veronii* by 16S rRNA gene sequencing (Figure 2).

## 4. Discussion

*Aeromonas* spp. are among the main causes of bacterial hemorrhagic septicemia associated with outbreaks and high mortality in global [36] and Brazilian tilapia farming [37]. Opportunistic pathogens such as *Aeromonas* spp. could serve as important ARG vectors for clinically relevant fish pathogens that share the same farming sites [21]. However, identification at the species level has not been an easy issue because they are cosmopolitan bacteria of the aquatic environment [8] and Nile tilapia microbiota [38]. In fact, colonies suggestive of *Aeromonas* spp. frequently appear in bacteriological cultures of clinical cases of MAS in Brazil but are routinely not properly identified. Current diagnostic techniques have increased the probability of making a specific diagnosis at the species level, although the clinical and pathological aspects of the field should always be considered to evaluate possible causal relationships. Furthermore, since motile *Aeromonas* are zoonotic bacteria, Rodrigues et al. [39] suggest that fish producers routinely use laboratory diagnostic techniques to evaluate the presence/absence of this pathogen in order to ensure public health.

To our knowledge, this study confirmed for the first time that the causative agent of the outbreak of clinically suspected MAS disease in net-cage-farmed Nile tilapia in Brazil was *A. veronii*. However, *A. hydrophila* and *A. jaudiae* have been previously described to be associated with mass mortality in farmed Nile tilapia in Brazil [4]. In Brazilian tilapiculture, there has not yet been a comprehensive study on the identification of motile *Aeromonas* species, but a recent study described that out of 124 isolates of *Aeromonas* spp. from tilapia farmed in Malaysia, *A. dhakensis* was the most frequent (43%), followed by *A. veronii* (22%), *A. hydrophila* (20%), *A. caviae* (8%), and *A. jandaei* (7%) [40].

In the present study, naturally and experimentally infected Nile tilapia exhibited clinical signs and gross pathology commonly seen in *A. veronii* infections [7,9,10,11]. The results of our experimental challenge showed significant differences in the mortality rates achieved in the groups of fish infected with high doses (1.2 × 10^7^ and 1.2 × 10^8^) with those infected at low and medium doses (1.2 × 10^4^ to 1.2 × 10^6^), which coincides with what has been previously described for *A. veronii* [7] and *A. jandaei* [4] infection in tilapia. The progression of the mortality rate in this study, characterized by a rapid onset (before 24 h), is consistent with that described for Nile tilapia experimentally infected with *A. veronii* [7], but also with *A. jandaei* [4].

However, the low mortality and slight histopathological changes in the liver, kidney, and spleen of surviving fish at the end of the trial recorded in fish from the medium and low-dose inoculated groups might suggest that low *A. veronii* loads in water and/or in fish not subjected to stressful or immunosuppressive conditions, e.g., in this study, natural co-infection with ISKNV under field conditions, might not be an important cause of mass mortality episodes under field conditions. Natural outbreaks of *A. veronii* have also been previously described in farmed tilapia in the presence of co-infections with other prevalent bacterial and viral pathogens [41]. Our results suggest that fish exposed to a sublethal dose of *A. veronii* could activate a protective immune response that would prevent an outbreak associated with mass mortality. These findings agree with those described by Dong et al. [7] following primary and secondary infection with *A. veronii*. A recent study conducted on dark sleeper fish experimentally infected with *A. veronii* [19] could shed some light on how this bacterium would modulate the innate and adaptive immune responses in Nile tilapia.

The development of resistance to antibiotics is the quintessential One Health issue [42], so it is essential to always safeguard their reasonable use. In fact, *Aeromonas* spp. is at the interface of all components of One Health and represents a solid example to explain its approach, from economic losses in aquaculture to the challenges related to antibiotic-resistant bacteria selected from the environment [43]. In this study, although *A. veronii* showed resistance to OTC, ENR, and AML, the bacterium was highly sensitive to FFC. Therefore, the producer was recommended to change the ineffective therapy based on AML for an FFC-medicated feed therapy (10 mg/kg), which quickly controlled the outbreak. Recently, Sakulworakan et al. [44] showed that *A. veronii* strains isolated from farmed tilapia in Thailand exhibited a multidrug resistance phenotype which was related to the presence of 20 multiple antimicrobial resistance genes (16 genes were shared among the *A. veronii* population). All tilapia isolates were susceptible to FFC (0%) but resistant to AML (100%) and ENR (17%), while OTC (67%) resistance was the second most dominant. Similarly, *Aeromonas* isolates from Malaysia showed resistance to AML (65%) and OTC (15%), but not to levofloxacin (0%) [40].

Our results are not only consistent with the phenotypic resistance patterns of *A. veronii* described in this study but also with the expression of the *qnrS2* gene refers to the plasmid-mediated quinolone resistance protein. In addition, we have also confirmed in the *A. veronii* strain described here, the presence of the previously described ColE as a plasmid of the *qnrS* gene [45]. These results correlate with that described by Hayatgheib et al. [46] on *Aeromonas* spp. isolated from farmed rainbow trout, and by Yang et al. [47] specifically on *A. veronii* strains isolated from farmed channel catfish. Furthermore, *tetA*-producing *Aeromonas spp* has been described in different environments from different countries [43,48,49]. Sakulworakan et al. [43] have recently described a resistome analysis of *A. veronii* isolates that presented several ARGs, including *qnrS* and *tetA*. Likewise, Woo et al. [49] reported *A. hydrophila* and *A. veronii* isolates displaying multidrug-resistant phenotypes harboring different ARGs such as *tetA, tetD, tetE, qnrB,* and *qnrS*, among others. In aquaculture, tetracycline has been used to treat bacterial infections in fish and other aquatic organisms [50]. However, the fish gut does not properly absorb oxytetracycline that must be administrated at high doses [51] and changes in gut bacterial community composition in rainbow trout have been described [52]. Taken together, these results provide evidence that conventional antimicrobials routinely used in aquaculture are losing their efficacy and that there is a plausible possibility that the acquisition of resistance is plasmid-mediated. Moreover, this evidence would suggest that *Aeromonas* could be used as an indicator of antimicrobial susceptibility in aquatic ecosystems.

Overall, our results suggest that *A. veronii* may represent a health risk in farmed tilapia and should be kept under surveillance under field conditions [53], especially at stages of the production cycle when fish are exposed to co-infections and/or stress factors such as high stocking density, excessive presence of organic matter in the water, high fungal and microalgae loads, high levels of ammonium and nitrite, and/or sudden changes in temperature.

## 5. Conclusion

To our knowledge, this study would constitute the first report of highly pathogenic and multidrug-resistant *A. veronii* associated with outbreaks and high mortality rates in tilapia farmed in commercial net cages in Brazil. This finding, together with other bacterial pathogens recently described in Brazilian tilapiculture, would highlight the importance of designing and implementing disease surveillance programs based on the correct identification of pathogens to facilitate assertive and timely decision-making at the farm production level, as well as how to establish the reasonable use of antimicrobials and an optimal vaccination strategy for control.

## Figures and Tables

**Table 1:** Primers, annealing temperature, and fragment size for screening genes encoding β-lactamases and plasmid-mediated quinolone resistance determinants and for tetracycline.

Gene	Primers	Sequences (5′ - 3′)	Annealing Temperature (°C)	Amplicon (pb)	Reference
*bla*_CTX-M-1_	CTX-M1 f CTX-M1 r	AAAAATCACTGCGCCAGTTC AGCTTATTCATCGCC ACGTT	52	415	[28]
*bla*_CTX-M-2_	CTX-M2 f CTX-M2 r	CGACGCTACCCCTGCTAT CCAGCGTCAGATTTTTCAGG	52	552	
*bla*_CTX-M-8_	CTX-M8 f CTX-M8 r	TCGCGTTAAGCGGATGATGC AACCCACGATGTGGGTAGC	52	666	
*bla*_CTX-M-9_	CTX-M9 f CTX-M9 r	CAAAGAGAGTGCAACGGATG ATTGGAAAGCGTTCATCACC	52	205	
*bla*_CTX-M-25_	CTX-M25 f CTX-M25 r	GCACGATGACATTCGGG AACCCACGATGTGGGTAGC	52	327	
*bla*_NDM_	IsoCarba_NDM f IsoCarba_NDM r	ACTTGGCCTTGCTGTCCTT CATTAGCCGCTGCATTGAT	66	603	[29]
*bla*_VIM_	IsoCarba _VIM f IsoCarba_VIM r	TGTCCGTGATGGTGATGAGT ATTCAGCCAGATCGGCATC	66	437	
*bla*_IMP_	IsoCarba_IMP f IsoCarba _IMP r	ACAYGGYTTRGTDGTKCTTG GGTTTAAYAAARCAACCACC	66	387	
*bla*_KPC_	IsoCarba_KPC f IsoCarba_KPC r	TCGCCGTCTAGTTCTGCTGTCTTG ACAGCTCCGCCACCGTCAT	66	353	
*bla*_OXA-48_	IsoCarba_OXA-48 f IsoCarba_OXA-48 r	ATGCGTGTATTAGCCTTATCG CATCCTTAACCACGCCCAAATC	66	265	
*qnrA1* a *qnrA6*	QnrAm-f QnrAm-r	AGAGGATTTCTCACGCCAGG TGCCAGGCACAGATCTTGAC	54	580	[30]
*qnrB1* a *qnrB6*	QnrBm-f QnrBm-r	GGMATHGAAATTCGCCACTG TTTGCYGYYCGCCAGTCGAA	54	264	
*qnrS1* a *qnrS2*	QnrSm-f QnrSm-r	GCAAGTTCATTGAACAGGGT TCTAAACCGTCGAGTTCGGCG	54	428	
*qnrD*	QnrD-f	CGAGATCAATTTACGGGGAATA	62	581	[31]
	QnrD-r	AACAAGCTGAAGCGCCTG			
*qnrC*	QnrC-f	GGGTTGTACATTTATTGAATC	50	447	[32]
	QnrC-r	TCCACTTTACGAGGTTCT			
*ColE plasmid*	ColE-f	GTTCGTGCATACAGTCCA	60	187	[33]
	ColE-r	GGCGAAACCCGACAGGACT			
*tetB*	tetB f	TTGGTTAGGGGCAAGTTTTG	55	659	[34]
	tetB r	GTAATGGGCCAATAACACCG			
*tetC*	tetC f	CTTGAGAGCCTTCAACCCAG		418	
	tetC r	ATGGTCGTCATC TACCTGCC			
*tetD*	tetD f	AAACCATTACGGCATTCTGC		787	
	tetD r	GACCGGATACACCATCCATC			
*tetA*	tetA f	GCTACATCCTGCTTGCCTTC	55	210	
	tetA r	CATAGATCGCCGTGAAGAGG			
*tetE*	tetE f	AAACCACATCCTCCATACGC		278	
	tetE r	AAATAGGCCACAACCGTCAG			

**Table 2:** Antibiotic sensitivity pattern and MIC values obtained for *A. veronii* against different antimicrobials.

Antibiotic Drugs	Classification Ranges (mm)		Inhibition Zone (mm)	Result	CIM (µg/mL)
	Wild-Type (WT)	Non-Wild Type (NWT)			
Oxitetracycline (OTC)	≥23	≤22	18	NWT	32
Enrofloxacin (ENR)	≥32	≤31	18	NWT	4
Amoxycillin (AML)	*	*	10	*	≥256
Florfenicol (FFC)	≥25	≤24	35	WT	≤0.5

* Aeromonas sp. are intrinsically resistant to beta-lactams.

## Data Availability

Data supporting reported results can be found at National Center for Biotechnology Information (NCBI), US National Library of Medicine (NIH) website under the accession numbers OR227081-227084 (16S rRNA), OR236713-236716 (rpoB), and OR236717-236720 (gyrB).
